# SHEAR saliva collection device augments sample properties for improved analytical performance

**DOI:** 10.1002/btm2.10490

**Published:** 2023-01-17

**Authors:** Shang Wei Song, Rashi Gupta, Niharika Jothilingam, Xinlei Qian, Yue Gu, V Vien Lee, Yoann Sapanel, David Michael Allen, John Eu Li Wong, Paul MacAry, Dean Ho, Agata Blasiak

**Affiliations:** ^1^ The N.1 Institute for Health (N.1), National University of Singapore Singapore Singapore; ^2^ Life Sciences Institute, National University of Singapore Singapore Singapore; ^3^ Department of Microbiology and Immunology Yong Loo Lin School of Medicine, National University of Singapore Singapore Singapore; ^4^ The Institute for Digital Medicine (WisDM), Yong Loo Lin School of Medicine, National University of Singapore Singapore Singapore; ^5^ Department of Medicine Yong Loo Lin School of Medicine, National University of Singapore Singapore Singapore; ^6^ Division of Infectious Diseases National University Hospital Singapore Singapore; ^7^ Department of Haematology‐Oncology National University Cancer Institute, National University Hospital Singapore Singapore; ^8^ Department of Biomedical Engineering College of Design and Engineering, National University of Singapore Singapore Singapore; ^9^ Department of Pharmacology Yong Loo Lin School of Medicine, National University of Singapore Singapore Singapore

**Keywords:** antigen rapid test, infectious diseases, point‐of‐care, saliva collection device, saliva‐based diagnostic, sample homogenizer, SARS‐CoV‐2

## Abstract

Despite being a convenient clinical substrate for biomonitoring, saliva's widespread utilization has not yet been realized. The non‐Newtonian, heterogenous, and highly viscous nature of saliva complicate the development of automated fluid handling processes that are vital for accurate diagnoses. Furthermore, conventional saliva processing methods are resource and/or time intensive precluding certain testing capabilities, with these challenges aggravated during a pandemic. The conventional approaches may also alter analyte structure, reducing application opportunities in point‐of‐care diagnostics. To overcome these challenges, we introduce the SHEAR saliva collection device that mechanically processes saliva, in a rapid and resource‐efficient way. We demonstrate the device's impact on reducing saliva's viscosity, improving sample's uniformity, and increasing diagnostic performance of a COVID‐19 rapid antigen test. Additionally, a formal user experience study revealed generally positive comments. SHEAR saliva collection device may support realization of the saliva's potential, particularly in large‐scale and/or resource‐limited settings for global and community diagnostics.

**Abbreviations**
ART: antigen rapid test
BCA: bicinchoninic acid
CV: coefficient of variation
DTT: dithiothreitol
LOD: limit of detection
POC: point‐of‐care
SCD: saliva collection device

## 1 INTRODUCTION

Saliva is receiving increasing attention as an analytical sample for biomonitoring, particularly in the context of the COVID‐19 pandemic, in order to improve diagnostic and screening throughput, among other factors. Saliva‐based diagnostic testing for detection of SARS‐CoV‐2 virus has been progressively adopted in the United States, Australia, and Singapore.^1^, ^2^, ^3^ The simple and noninvasive collection methods of saliva enable self‐testing as sampling can be conducted safely without the supervision of trained personnel, reducing the risk of transmission of the pathogen to healthcare workers. Self‐sample collection and testing further allow for an increase in testing capacity and the potential for earlier identification and isolation of pre‐symptomatic individuals. These are important considerations for all regions where initial outbreaks may strain testing resources and/or low‐ and middle‐income countries (LMICs). Beyond infectious disease testing, saliva has shown promise for other diagnostic applications due to the plethora of analytes such as hormones, enzymes, and antibodies that it contains.^4^

Beyond the analytes of current diagnostic interest, saliva contains a high concentration of glycoproteins, especially mucin.^4^, ^5^ Glycoproteins protect the buccal epithelium from chemicals, microbes and wear‐and‐tear but make saliva a difficult analytical matrix due to the formation of high viscosity pockets,^6^ which complicate accurate sample processing (e.g., pipetting of accurate volumes) and automation.^7^, ^8^, ^9^, ^10^, ^11^ Additionally, salivary mucin networks form complexes with some of the proteins,^12^ potentially reducing their availability for detection. Furthermore, the pockets of viscosity result in nonuniform physical properties and nonuniform analyte distribution within the sample contributing to analytical variability. The current methods to homogenize biofluids include mechanical and chemical approaches, each with its own benefits and disadvantages. For example, a traditional mechanical method involves exposing the sample to cycles of freeze‐thawing. While this approach is cost‐effective and does not require extensive laboratory equipment or reagents, it is time consuming and multiple cycles of freeze‐thawing can alter the detection of analyte.^13^ A new mechanical homogenization method was proposed using magnetic rods.^11^ While faster, it requires a centrifuge and laboratory equipment, and is not suitable for small volumes. Chemical methods such as mucinase^14^ and dithiothreitol (DTT),^15^, ^16^ are much more popular, and were shown to degrade mucin, lowering sample's viscosity. While relatively fast (<1 h incubation) and not laboratory equipment‐extensive, they require a careful balance between the dissociation of the glycoprotein bonds and the retention of the 3D conformational analyte properties that might be the basis of its detection.^17^ Additionally, the reagents can be expensive and might have a limited availability ‐ the features that can preclude rapid deployment and scale up of diagnostic operations in the case of a local or global health emergency, such as the COVID‐19 pandemic.

In this study, we describe and evaluate the SHEAR saliva collection device (SCD), a safe and simple point‐of‐care (POC) mechanical sample homogenization device made up of three main components: a soft foldable funnel, a shearing filter and a collection tube. We report a reduction in viscosity with improvement in uniformity and an increase in detected total protein concentration in saliva samples processed with the SHEAR SCD due to the mechanical shearing of saliva by the shearing filter. This achieved sample homogenization is comparable to conventional saliva processing methods: freeze‐thawing, centrifugation, and chemical homogenization with DTT. Using rapid antigen testing (ART) with the SARS‐CoV‐2 nucleocapsid protein, we demonstrate that processing of saliva with SHEAR SCD improved diagnostics performance. Furthermore, the wide and soft funnel conforms to and fully covers the donor's mouth reducing the risk of others being exposed to the pathogen during saliva donation. Our findings from backflow and food particle tests display that the unique shearing filter also limits the backflow of saliva and reduces the size and count of food particles, enhancing user safety through prevention of spillage and ingestion of the content in the collection tube and mitigating potential interference with detection assay due to particle contaminant. Lastly, we also show SHEAR SCD is easy to use as assessed in a formal, qualitative user study. Together, these findings point out to the functional capability and user friendliness of SHEAR SCD for the collection and processing of saliva samples, especially for SARS‐CoV‐2 testing in the POC setting.

## 2 RESULTS

### 2.1 SHEAR SCD

**FIGURE 1:**
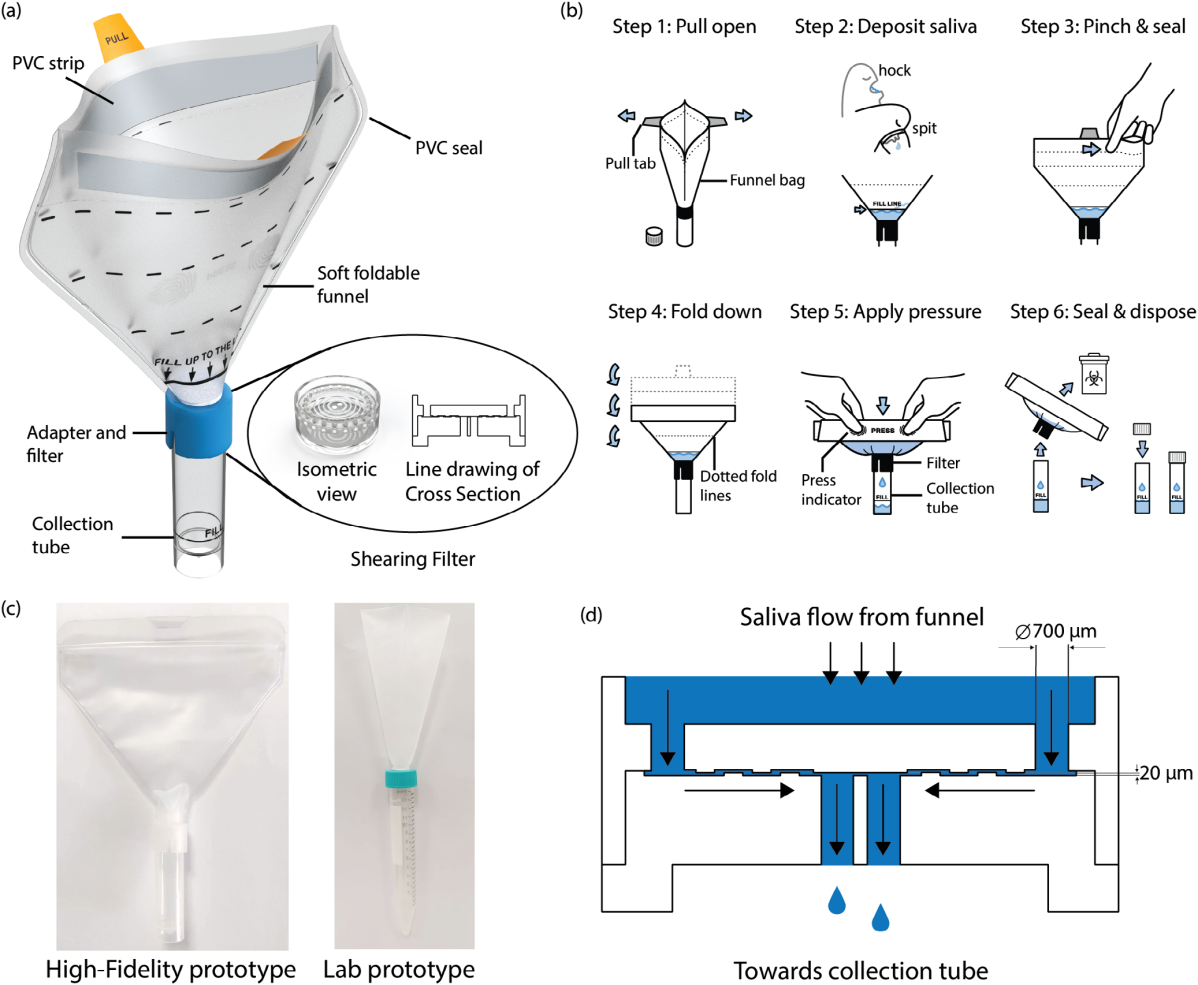
SHEAR SCD product design and instruction for use. (a) SHEAR SCD with labeled components. (b) Graphic of the instruction for use of SHEAR SCD. (c) Photo of the early lab prototype of SHEAR SCD. (d) Illustration for the flow of saliva through the shearing filter.

The advent of the COVID‐19 pandemic resulted in a sudden surge in demand for widespread SARS‐CoV‐2 testing and supply chain bottlenecks amplified the shortage of essential materials for diagnostic tests (e.g., nasopharyngeal swabs, reagents, etc). The SHEAR SCD was designed in response to rigid funnel shortages for collecting saliva, and to address potential procedural bandwidth limitations with regards to sample processing, among other factors during the substantial ramp‐up timeframe required to mobilize large‐scale testing. The SHEAR SCD is made of three main components: a wide (130 mm) and soft foldable funnel, a shearing filter, and a collection tube (Figure 1a). SHEAR SCD saliva sample collection involves six steps (Figure 1b). First, the funnel is opened by pulling the pull tabs and then a saliva sample is deposited. Once enough saliva is collected in the funnel (above the 1 ml fill line), the funnel is sealed, folded, and subsequently squeezed to generate pressure to drive saliva through the shearing filter and into the collection tube. Once enough saliva is collected in the collection tube, the funnel is disconnected and disposed while the collection tube is closed with a screwcap. The SHEAR SCD underwent product design cycles for an enhanced user experience and improved manufacturability. The high‐fidelity prototypes, used for all testing in this study were manufactured through injection molding techniques under ISO13485 conditions. The early lab prototype (before the redesign process) shown in Figure 1c was constructed within a week with readily available materials and technologies. The filters were 3D printed using an inert and rigid photopolymer with a liquid resin printer, and piping bags, commonly used for baking purposes, were modified to construct the soft funnel. A hole was bored into a screwcap of a 15 ml centrifuge tube to house the filter, and the centrifuge tube acted as the collection tube.

#### 2.1.1 Mechanical saliva shearing principles of SHEAR SCD

Mechanical shearing of saliva occurred at the shearing filter. The folding and squeezing of the device's funnel generated the pressure to propel saliva through a sequence of abruptly narrowing and expanding passages. First, the sample was pushed into multiple, round openings with the diameters of 700 μm located at the bottom of the funnel and at the top of the filter (Figure 1d). Subsequently, the sample traveled between two textured substrates before flowing into the collection tube via a pair of round openings with the diameters of 700 μm. The textured substrates created a narrow, meandering passage, with a height of the gap as low as 20 μm. The pressure combined with the reduction in the cross‐sectional area and meandering passage generated a shear stress acting on the sample within the filter (Figure 1d). We recorded a maximum pressure of 0.53 ± 0.11 kg/cm^2^ required to be generated in the funnel for successful processing of a saliva sample with SHEAR SCD.

### 2.2 SHEAR SCD characteristics for saliva collection

**FIGURE 2:**
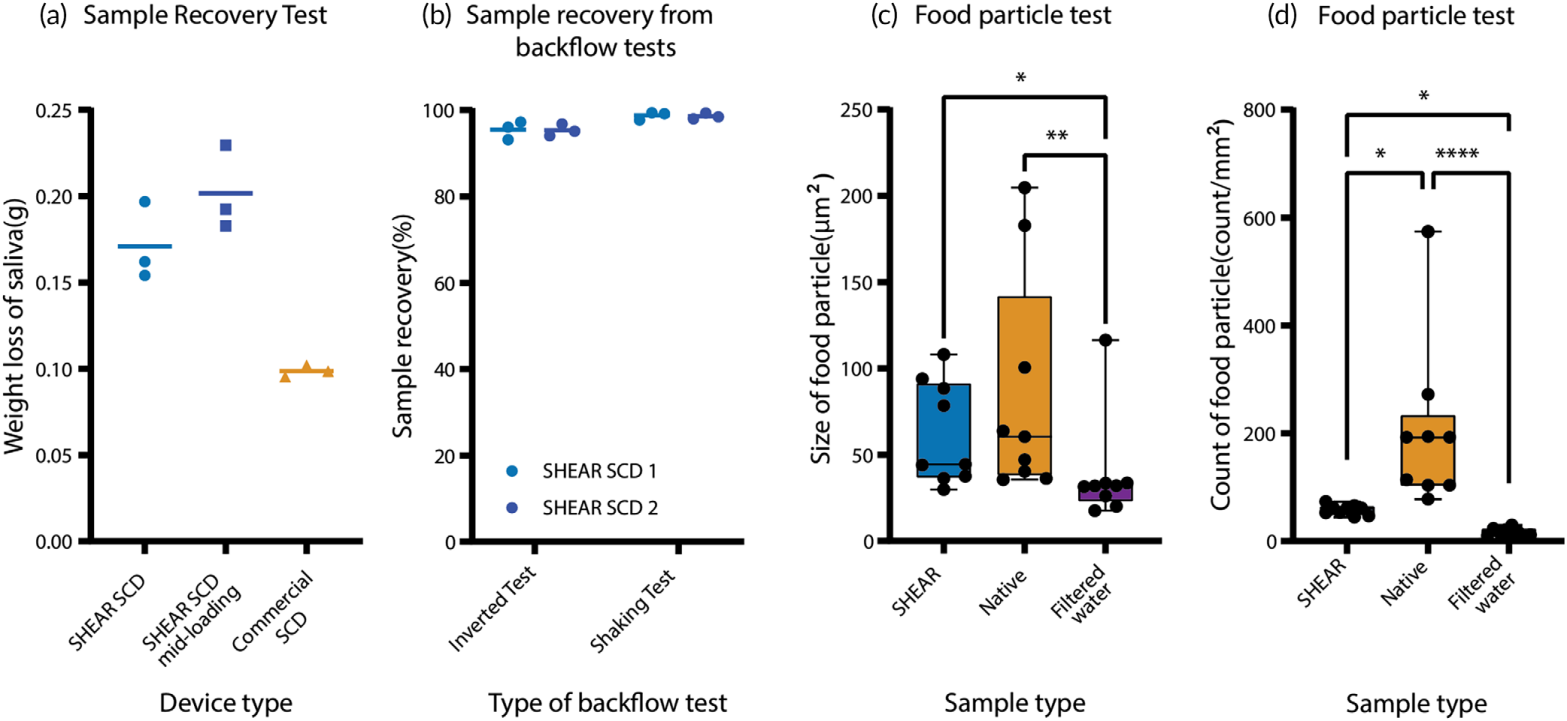
Sample recovery, backflow and food particle test results. (a) Weight loss of saliva from sample recovery testing of the SHEAR SCD (*n* = 3), the SHEAR SCD with sample loading in the middle of the funnel (*n* = 3), and the commercial SCD (*n* = 3). (b) Sample recovery of saliva samples from backflow tests; inverting test and shaking test, two SHEAR SCDs (SHEAR SCD 1 and SHEAR SCD 2, *N* = 3). (c) Size of food particles found in SHEAR(SHEAR SCD‐processed sample), native (native sample), and filtered water in the food particle test. (d) Count of food particles found in (count/mm^2^) of SHEAR (SHEAR SCD‐processed sample), native (native sample), and Filtered water in the food particle test. *N* = number of technical replicates, *n* = number of SCDs. Lines in (a and b) represent the mean value. Whiskers represent maximum and minimum values and the box represents the median value, 25th and 75th percentile (c and d). ****p < 0.0001, ***p < 0.001, **p < 0.01, and *p < 0.05.

A sample recovery test was conducted to investigate the sample retention in the SHEAR SCD. When 2 g saliva samples were pipetted directly to the base of the funnel, the average weight loss of saliva in SHEAR SCD and commercial SCD were 0.1710 ± 0.0227 g and 0.0986 ± 0.0034 g, respectively (Figure 2a). When saliva was pipetted to the middle of the SHEAR SCD funnel the weight loss was 0.202 ± 0.0246 g.

POC tests commonly include a buffer in the collection tube for enhanced analyte interfacing. Improper handling of a kit during the saliva donation process may lead to accidental ingestion of buffer medium from the collection tube. To test if the SHEAR SCD filter stops backflow of saliva (or saliva‐buffer mixture) from the collection tube, we performed two sample recovery tests with saliva present in the collection tube: shaking the tube with the SHEAR SCD in a horizontal position, and inverting the tube with the SHEAR SCD upside‐down. The average weight loss of saliva in the shaking test and the inverting test were 0.0268 ± 0.0151 g and 0.0932 ± 0.0307 g, respectively. This corresponded to the sample recovery rates of 98.8% and 95.4%, respectively (Figure 2b).

The presence of large food particles in saliva samples may introduce interference into the downstream analytical tests. Food particle analysis was conducted to test the efficacy of the SHEAR SCD’s shearing filter in trapping particles and reducing contamination. The median size and count of food particles found in SHEAR SCD‐processed samples were 44.38 μm^2^ (95% Confidence Interval [CI], 36.38–93.86) and 60.86 count/mm^2^ (95% CI, 46.07–64.84), respectively—a 36.1% reduction in size and a 215.8% statistically significant reduction in count as compared to the native samples before processing (Figure 2c,d).

### 2.3 SHEAR SCD enables saliva homogenization and analyte release

**FIGURE 3:**
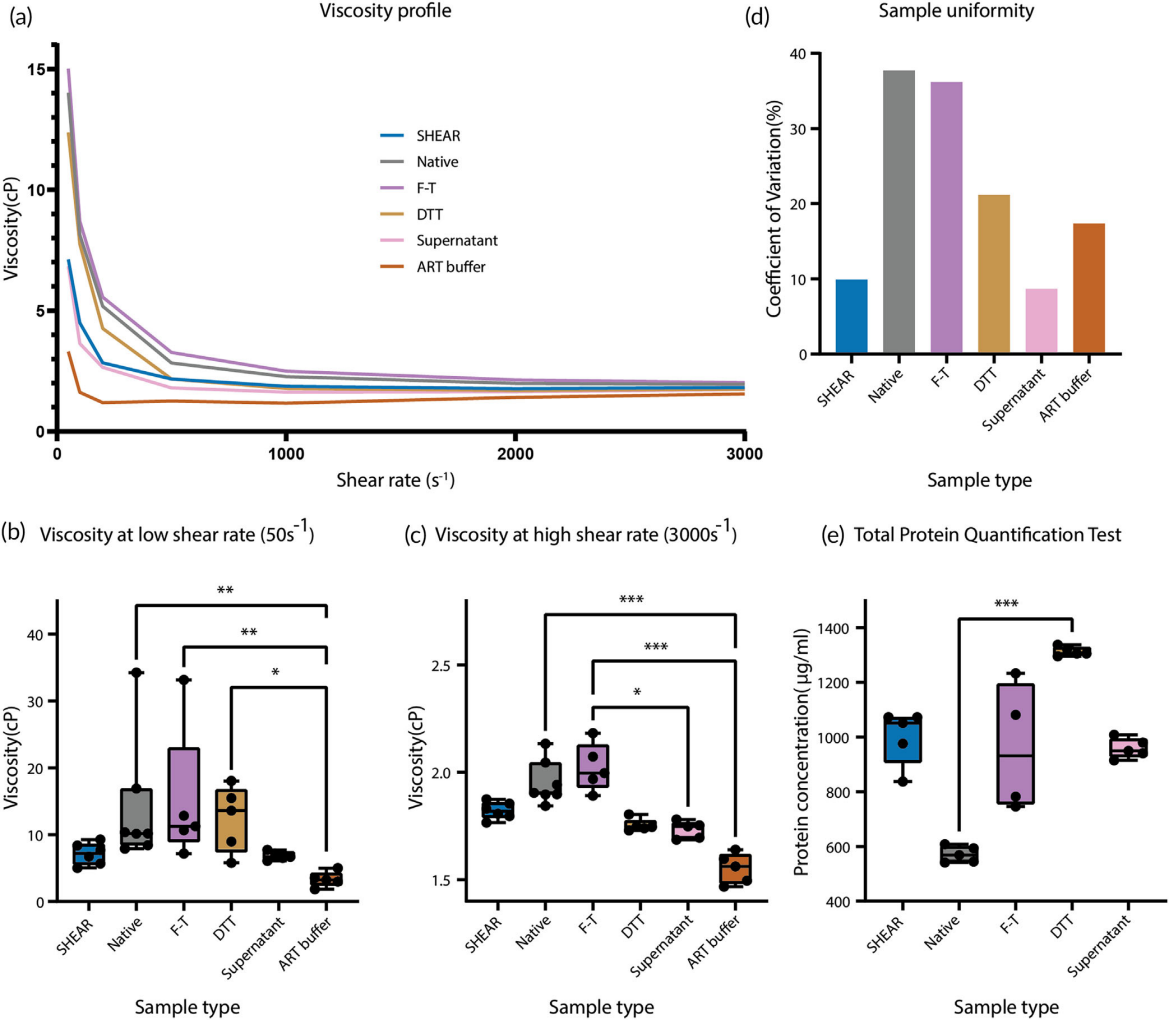
Viscosity and total protein concentration of saliva samples. (a) Viscosity profile of fluid samples measured with rheometer: SHEAR (SHEAR SCD‐processed saliva, *N* = 6), native (native saliva, *N* = 7), F‐T (freeze‐thawing‐processed saliva, *N* = 5), DTT (DTT‐processed saliva, *N* = 5), supernatant (centrifuged‐processed saliva, *N* = 5), and ART buffer (*N* = 5) over a shear range from 50 to 3000 s^−1^. (b) Viscosity of the fluid samples measured with rheometer at the low shear rate: 50 s^−1^. (c) Viscosity of the fluid samples measured with rheometer at the high shear rate: 3000 s^−1^. (d) Sample uniformity of the samples measured with rheometer over the shear range from 50 to 3000 s^−1^. (e) Estimation of total protein in samples processed with SHEAR SCD (SHEAR SCD‐processed saliva, *N* = 5), native (native saliva, *N* = 5), F‐T (freeze‐thawing‐processed saliva, *N* = 4), DTT (DTT‐processed saliva, *N* = 5), and supernatant (Centrifuged‐processed saliva, *N* = 5). *N* = number of technical replicates. Whiskers represent maximum and minimum values and the box represents the median value, 25th and 75th percentile (b, c, and e). ***p < 0.001, **p < 0.01, and *p < 0.05.

To assess the sample processing performance of the SHEAR SCD, the viscosity of SHEAR SCD‐processed saliva samples was measured across a shear range from 50 to 3000 s^−1^ and compared to saliva samples treated with other, conventional saliva processing methods and an ART buffer solution. The saliva samples and ART buffer solution exhibited a non‐Newtonian, shear thinning behavior (Figure 3a). While no statistically significant differences in saliva viscosity were detected between SHEAR SCD‐processed saliva and all measured samples, at the low shear rate (50 s^−1^) the viscosity of SHEAR SCD‐processed saliva was lower (7.20 cP [95% CI, 5.02–9.28]) than native, freeze‐thawing, and DTT processed saliva, but higher than that of Supernatant and ART buffer samples (Figure 3b). Except for DTT‐processed saliva, a similar trend of viscosity differences between SHEAR SCD processed and all measured samples was observed at the high shear rate (3000 s^−1^; Figure 3c). Additionally, SHEAR SCD‐processed saliva demonstrated an enhanced uniformity of viscosity, with coefficient of variation (CV) lower than all, but except the Supernatant samples (Figure 3d). Similar homogenization effects were observed in saliva samples with higher viscosity when processed with SHEAR SCD (Supporting Information Results, Figure S6). Lastly, SHEAR SCD‐processed saliva had a total protein concentration of 1051 μg/ml (95% CI, 837.7–1072.3), which was higher than native, freeze‐thawing, and supernatant samples, but lower than DTT‐processed saliva (Figure 3e).

### 2.4 SHEAR SCD preprocessing affects the diagnostic performance of an ART

**FIGURE 4:**
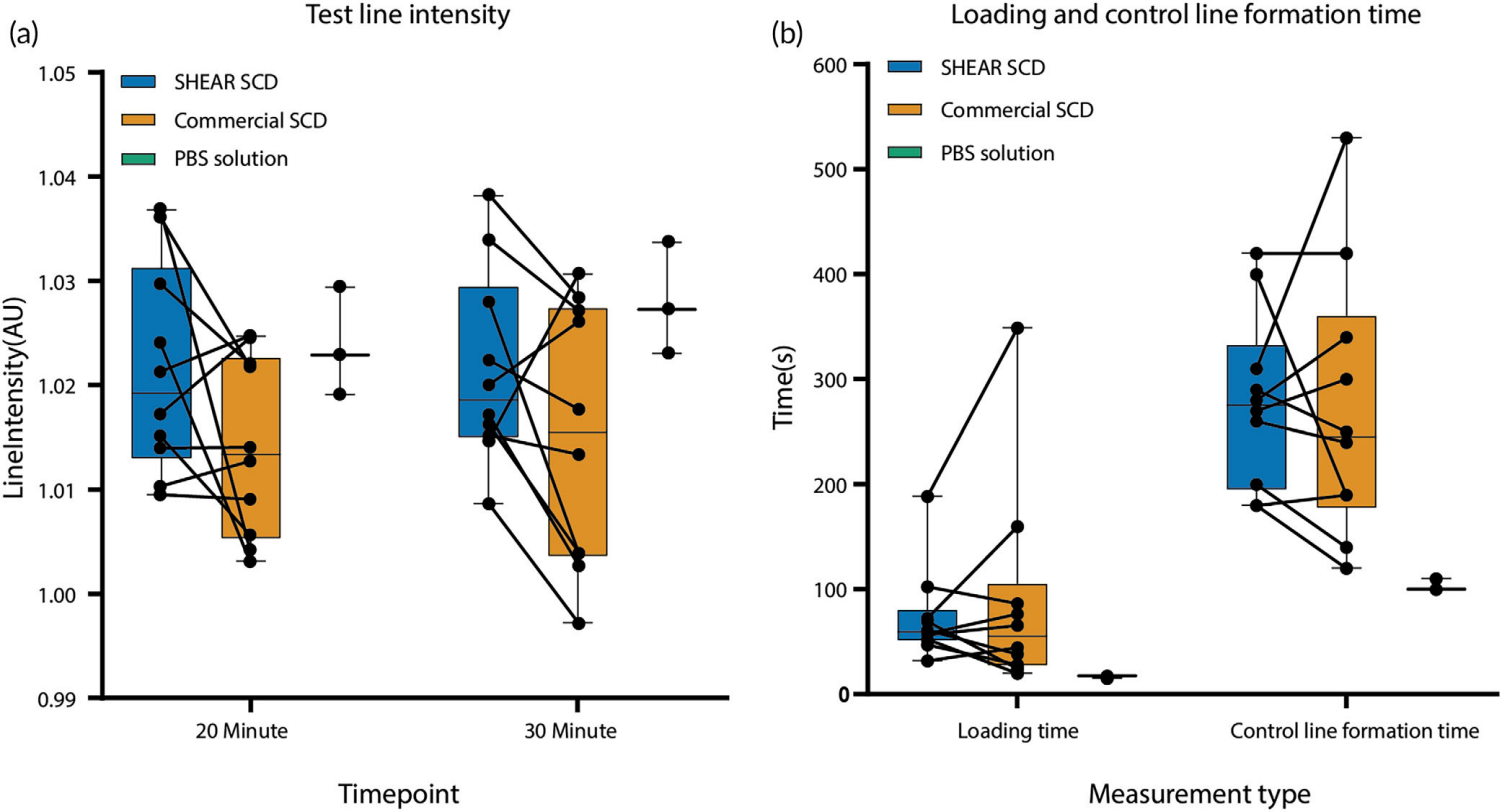
Test line intensity, loading time and control line formation time. (a) Test line intensity for SHEAR SCD‐processed saliva (*N* = 10), Commercial SCD‐processed saliva (*N* = 10), and PBS solution (*N* = 3) at 20‐ and 30‐min timepoints. (b). Loading time and control line formation time of SHEAR SCD‐processed saliva (*N* = 10), Commercial SCD‐processed saliva (*N* = 10) and PBS solution (*N* = 3). *N* = number of technical replicates. Whiskers represent maximum and minimum values, the box represents the median value, 25th and 75th percentile and the line represent the pairwise comparison between SHEAR SCD‐processed saliva and Commercial SCD‐processed saliva. No statistical difference was detected with Wilcoxon signed‐rank test at *α* = 0.05.

To investigate the effects of improved biophysical properties of saliva on an ART performance, paired saliva samples—processed with SHEAR SCD and commercial SCD—were spiked with SARS‐CoV‐2 nucleocapsid protein near the limit of detection (LOD) concentration level and tested with a commercial SARS‐CoV‐2 ART kit. The presence of a filtered out yellow residue was detected in the sample well of the ART kit for several individuals, with less of that residue detected in the paired, SHEAR SCD‐preprocessed samples (Figure S1). We measured the change in the intensity of the background after the passing of the sample, and the relative intensities of the test and control lines after 20 and 30 min from dropping the samples into the sample well of an ART cassette (Video S1). While there was no statistically significant difference in the intensities of the background and the lines between the two SCD types at either of the timepoints, the samples processed with the commercial SCD led to the formation of a visible test line for 6/10 individuals and SHEAR SCD improved that result by facilitating the test line formation in 9/10 samples from the same individuals (Figure 4a). No correlation was detected between the change in the background intensity and the relative intensity of the test line.

Additionally, we investigated the effects of SHEAR SCD on the temporal dynamics of sample loading and line formation. There was no statistically significant difference in the median loading time between the two groups (59.36 vs. 55.18 s, for the SHEAR SCD and the commercial SCD sample group, respectively). However, SHEAR SCD sample preprocessing decreased the variation in the loading time by 2 times—with a CV of 0.75 versus 1.82 in the SHEAR SCD and commercial SCD test groups, respectively. For comparison, the median loading time of the control samples (PBS; *N* = 3) was 17.5 s with CV of 0.08. We further assessed the time required for the sample after loading to pass the membrane assay across the result window and lead to the formation of the control line. The time required to form the control line was not statistically different between the two groups (3.25 min and CV of 0.42 vs. 3 min and CV of 0.31 in the SHEAR SCD and commercial SCD sample group, respectively) but was twice of that in the control group (1.5 min with CV of 0.06) (Figure 4b). Lastly, no correlation was found between liquid migration speed and line intensity for the test and control lines (*R* ^2^ = 0.035 and 0.095, respectively) (Figure S2).

### 2.5 SHEAR SCD is user friendly

Qualitative interviewing is a commonly used method for evaluation of the device usability as it is able to offer insights into user's choice, thoughts and feelings. Usability evaluation through interviewing and qualitative analysis has been reported to be helpful for augmenting medical device design, safety, and effectiveness.^18^ Hence, we performed a formal behavioral study to gauge participants attitude toward processing of their own saliva with SHEAR SCD.

Fifteen healthy individuals participated in the user study between February and June 2022. There were eight female and seven male participants with a median age of 27 years (range 23–60 years). The participants were provided with an instructional video (n1labs.org/shear-scd) and were asked to donate and process their saliva with SHEAR SCD. All participants were able to complete the processing of saliva with SHEAR SCD without additional help. Participants were then asked to donate saliva into a standard, commercial rigid funnel and then participated in a semi‐structured interview. Data saturation was achieved with 15 participants and no new themes emerged after participant 11.

Usability and functionality of the SHEAR SCD was one of the main themes that emerged from the interviews. Participants commented positively toward SHEAR SCD and provided some suggestions for improvements. 93.3% of the participants responded favorably toward the usability of SHEAR SCD, specifically regarding the ease of use and straightforwardness of the SCD. Furthermore, all participants found that the instructions for use of SHEAR SCD to be simple, clear and easy to understand, which boosted their confidence in handling the SCD. According to Participant 6, “The [instructional] video really helped. I wouldn't have the confidence to use it if I have not watched the video.” Thirteen out of fifteen users commented positively on the additional functionality offered by the SHEAR SCD. The participants described an increased comfort level, ease of saliva donation and increased perceived safety level due to the large funnel size. In addition, two participants commented on the enhanced suitability of SHEAR SCD for elderly due to the large funnel size. Eleven participants highlighted the more simplistic nature of the commercially available SCD. For instance, Participant 10 shared, “The [commercial device] was simpler, but the [SHEAR SCD] actually has a function.” The sparse difficulties reported by participants were mainly attributed to the presence of a push back of the saliva into the funnel during the squeezing and folding steps, lack of saliva level indicator (in contrast to its presence in the instructional video) and insufficient air trapping within the funnel. Lastly, one participant highlighted the challenges users with disability might encounter during the processing step of using the SHEAR SCD. An additional two themes that emerged were: saliva as a biological material for diagnostic tests and user's consideration for the adoption of diagnostic kit with SHEAR SCD (Supporting Information Results, Table S1).

## 3 DISCUSSION

### 3.1 SHEAR SCD fulfills basic requirements for saliva collection at scale

Safety and effectiveness are two essential components that need to be demonstrated for any medical device, including an SCD. While uncommon, several accidental exposures to buffer solutions from diagnostic test kits resulting in minor health outcome have been reported.^19^ In our backflow tests, over 95% of the saliva sample was retained in the collection tube even after inverting the SCD, demonstrating the SHEAR SCD filter's ability to mitigate the risk of spillage, which may lead to a lowered incidence of saliva sample and buffer solution exposure to the user. Furthermore, the 36.1% and 215.8% reduction in size and number of food particles found in the SHEAR SCD‐processed sample, may decrease the chances of particle interferences with diagnostic assays due to food debris.^20^

Transmission of respiratory viruses can occur through direct contact with aerosol and droplets containing virions^21^ released during sneezing and coughing.^22^ Similarly, the spitting and hocking actions during a saliva collection pose a risk of droplet expulsion and viral transmission. Furthermore, the incidence of saliva contamination on exterior surfaces of collection tube have been reported in a recent study.^23^ SHEAR SCD enables saliva collection with the user's mouth and nose covered by the funnel, which contains the dispersion of droplets within the SCD and prevents saliva leakage to the exterior surfaces of the SCD. The folding and sealing of the SHEAR funnel also reduce the risk of environmental contamination. While the wide funnel of the SHEAR SCD augments user's safety, it might have contributed to the increase in saliva retention observed in our sample recovery test. However, the sample recovery rate was still above 90%, which is sufficient to perform a follow up diagnostic assay.

### 3.2 SHEAR SCD improves the physical properties of saliva for diagnostics analysis

Saliva with high viscosity is challenging for both laboratory‐based testing and POC diagnostic applications involving lateral flow assay and paper‐based analytical devices. Saliva being a non‐Newtonian fluid, exhibits shear thinning behavior. Studies have shown that native movement of mouth such as swallowing and speaking corresponds to low shear rate of saliva^24^ and saliva is subjected to a high shear rate when it is flowing through small openings such as pipette tips.^25^ The observed reduction in saliva viscosity throughout the whole tested shear rate range, due to the mechanical shearing effects of the SHEAR SCD filter, can ease the pipetting procedures in laboratory‐based saliva testing reducing pipetting errors, the risk of environmental cross contamination and processing time required.^7^, ^8^, ^10^ No decrease in viscosity was observed for the freeze‐thawing‐processed saliva samples. This may be due to the absence of the subsequent centrifugation step reported previously.^9^, ^26^, ^27^ In a separate set of samples, we noted that the viscosity of supernatant was comparable to that of SHEAR SCD‐processed saliva, however, the implementation of SHEAR SCD for POC application might be more feasible since it does not require additional equipment.

Color formation in the BCA assay indicates number of peptide bonds and presence of specific amino acids.^28^ We observed an increase in total protein concentration in all processed saliva samples when compared to the native saliva sample. Breaking down mucopolysaccharide can potentially release analytes bound to the mucin networks.^11^ Specific to COVID‐19, a disruption of mucin networks may release SARS‐CoV‐2 spike protein bound to the sialic acid glycan end of mucins,^29^ and enhance virus detection. In our study, DTT‐processed saliva samples displayed the highest increase in protein concentration suggesting the greatest disruption of mucin networks. DTT is a commonly used reducing reagent for chemical homogenization of saliva samples and acts through splitting of disulfide bond between proteins. While effective, sample homogenization with DTT or other harsh chemical reagents may impose alterations in biochemical properties of the analytes, which may lead to their impaired detection. For example, sample homogenization with DTT has been shown to reduce the concentration of sputum myeloperoxidase, a potential biomarker for chronic obstructive pulmonary disease.^30^

### 3.3 SHEAR SCD affects ART testing

Saliva samples processed with SHEAR SCD demonstrated a mild improvement in the test line intensity. Previous studies suggest the amplification of the signal intensity of viscous samples with low flow rate across a lateral flow assay due to increased incubation time of the analyte at the test lines^31^ and an elevated reaction time at the conjugate pad.^32^ Several other studies corroborated the effects of viscosity on the flow rate of the liquid sample across a lateral flow assay.^33^, ^34^ In contrast, no correlation was found between sample flow rate and line intensity in our study. Instead, we hypothesize, that as the nucleocapsid protein at the fixed concentration was spiked into the liquid fraction of the saliva samples after they passed through the SCDs, it is plausible that the higher uniformity and lower viscosity of the SHEAR SCD‐processed samples allowed better dispersion of the analyte and the buffer and led to enhancing the analyte concentration within the sample dropped onto the ART's sample well to increase the test line intensity. A natural variation exists in analyte distribution in different fractions of saliva depending on salivary gland that produced it. Our results may indicate that homogenizing the sample can increase the band intensity through improving the analyte distribution. Of note, nucleocapsid protein was spiked into the saliva samples near the LOD concentration to represent the challenging detection cases in individuals with low viral load. The improved intensity and therefore visibility of the test line after sample preprocessing with SHEAR SCD reduced false negatives. Further investigations are needed to validate this result in a clinical setting.

The sample well and the membrane of an ART allow only the liquid fraction to pass through to interact with the antibody conjugated strips and form the lines. Accordingly, SHEAR SCD had no effect on temporal dynamics of the sample migration after loading. However, SHEAR SCD lowered the variation in the sample loading time, which facilitate identification of a universal readout period for the most reliable results by lowering the interindividual variation of saliva samples' biophysical properties.

### 3.4 SHEAR SCD offers an optimal user experience

Developing an engineering solution for a medical application, benefits from including all stakeholders in the co‐development process.^35^ Through interviews with the users, we sought to identify the attitudes toward SHEAR SCD, particularly as an additional step (folding the funnel) is expected from the saliva donor. In general, participants of the user study commented that SHEAR SCD was straightforward and easy to use, with simple and easy to understand instructions. Targeted refinements of SHEAR SCD based on the highlighted difficulties will benefit the usability and functionality for enhanced user‐experience. In our pressure testing, the pressure required to squeeze saliva through the filter (0.53 ± 0.11 kg/cm^2^) was below the pressure level that an average 70‐year‐old female (3.9–4.3 kg)^36^ and even an elderly individuals with unilateral thumb carpometacarpal (1.9–3.1 kg)^37^ generate as measured through pulp pinching. It is consistent with the findings from the user study, where no difficulties in pushing of saliva due to pressure required were reported. The positive reception of the users points to a potential of a wide adoption of SHEAR SCD.

### 3.5 Limitations

While offering multiple benefits, SHEAR SCD is not without limitations. The effect of mechanical shearing is limited, and even though no statistical significance was detected, the viscosity of SHEAR SCD‐processed saliva was higher than that of buffer solution used in the ART. The future developments may include combining SHEAR SCD with a mild chemical treatment to maximize homogenization while preserving the analyte. An experimental limitation is that in the total protein and rheology experiments saliva samples collected from multiple participants were pooled, as both experiments required more amount of saliva than what an individual can donate in a sitting contributing to a detected variation in the saliva viscosity. To lower its effects, saliva samples in all groups were gently vortexed for 1 s right before each test.

The ART kit in the experiments was used off label ‐ the saliva sample was not a validated specimen listed in the instruction for use. Also, spiking of SARS‐CoV‐2 nucleocapsid proteins into processed healthy saliva samples limits the evaluation of the effects of mechanical shearing on mucin networks, which can potentially release analytes trapped in that network. The benefits of SHEAR SCD for POC kits will require further testing, including in clinical trials with this study setting a foundation for such endeavor. Lastly, in the user study the median age of the participants was 27 years (ranging from 23 to 60 years) and only healthy individuals were recruited, hence the findings from the study maybe have limited generalizability to elderly, children and people with disabilities.

### 3.6 SHEAR SCD's relevance for saliva‐based clinical testing beyond COVID‐19

Saliva has an advantage over other diagnostic samples due to the easy, safe, and noninvasive nature of sample extraction. Similar to the responses recorded from participants in our user study, patients also cited these among reasons for which they prefer oral fluid sampling when given the choice.^38^ Furthermore, fear of self‐nasal swabbing may lead to shallower swabbing,^39^ potentially amplifying the probability of false‐negative results. The noninvasive nature of saliva collection may be greatly beneficial for broad deployment under epidemic and pandemic circumstances, but also in a routine care, especially for patients that require inpatient and/or outpatient longitudinal tests. These factors may potentially increase compliance with the testing regime, lowering the stress exerted on patients, and potentially enabling accurate remote monitoring.

As demonstrated in COVID‐19 diagnostics, POC testing has a potential for an early detection of the infection, which in turn allows earlier management options to be commenced and a greater likelihood for successful therapy. The benefits extend to other infectious diseases—for example, saliva‐based POC tests can be used for the diagnosis of HIV^40^ and Hepatitis C, where the presence of immunoglobulins can inform about the stage of the infection.^41^ Other infectious diseases, for example, Hepatitis B, mumps, and rabies, also has a potential to be detected in saliva.^42^ Beyond infectious diseases, salivary diagnostics are clinically applicable to monitoring drug abuse, hormone levels, and a range of disease markers. For example, saliva‐based monitoring of C‐reactive protein, a nonspecific inflammatory response factor, has a potential to assist in detection and tracking of inflammation,^43^, ^44^ enabling earlier detection of infection and supporting rapid decision making. Similarly, being able to monitor drug levels with saliva‐based POC testing may help reduce the number of invasive blood tests.^45^

While salivary diagnostics hold potential, they are not often used as the first line or stand‐alone tests. Broadly, deployed applications of saliva‐based POC testing are also not common. The challenges stem from minute quantities of the markers and sample variability.^46^ Improving the biophysical properties of saliva sample for diagnostics at the collection stage with SHEAR SCD offers a range of benefits and may potentially contribute to the broader realization of saliva's potential as a clinical sample beyond COVID‐19, especially in POC applications.

## 4 MATERIALS AND METHODS

### 4.1 Manufacturing method of SHEAR SCD

The SHEAR SCD was fabricated and assembled by an ISO 13485:2016 certified manufacturer (Forefront Medical Technology, Singapore). The soft funnel was made from die‐cutting and heat sealing of PolyVinyl Chloride sheets. The filter was manufactured with micro‐injection molding of Polypropylene (PP, K1P38AE) using MicroPower15t machine (Wittmann Battenfeld, Austria) and the adapters were constructed with injection molding of Polypropylene (PP, K1P38AE). Medical grade adhesive (Loctite 3921, USA) was used to bond the filter and the soft funnel to the adapter. Standard collection tubes were used.

### 4.2 Saliva collection method and IRB

Saliva samples were collected from 24 healthy volunteers aged between 21 and 65 years in accordance with protocol approved by the Institutional Review Board of National University of Singapore (NUS‐IRB‐2021‐15); written informed consent was obtained from each volunteer. Body temperature of each volunteer was taken prior to the study and any volunteers that exhibited acute respiratory infection symptoms were excluded. Volunteers were advised to avoid brushing of teeth and consumption of food and beverages for at least an hour before the collection of saliva. The volunteers were advised to donate deep throat saliva. All samples were collected between 09:00–11:00 and 13:00–14:00.

### 4.3 Filter performance experiments

#### 4.3.1 Food particulate test

Five milliliter of filtered water (Mili‐Q IQ 7000) was spiked with 0.1 g of chili flakes and added to the funnel of one SHEAR SCD and one common, commercially available SCD (MicroCollect™ Saliva Collection Device, CD Genomics, USA). The mixture collected in the collection tube of each SCD and a control solution—filtered water—were imaged on a glass slide at nine fixed points with a light microscope (4× magnification, ECLIPSE Ti‐S, Nikon, Japan). Particulate counting and size measurement were performed with ImageJ (National Institutes of Health, USA) (Figure S4).

#### 4.3.2 Characterization of backflow

In the first test, two SHEAR SCDs with 2 g of saliva in their collection tubes were placed on an orbital shaker (Orbital shaker OS‐20, Boaco, Germany) and shook at 100 rpm for 60 min. In the second test, two SHEAR SCDs with 2 g of saliva in their collection tubes were vertically inverted and placed on a retort stand for 60 min. The weight of the SCDs with and without the sample, as well as the weight of the collection tube before and after each test were recorded and used to characterize the sample backflow. Both tests were performed in triplicates.

#### 4.3.3 Sample recovery

Two gram of saliva were added and processed in SHEAR SCD or commercial SCD. The weight of the SCDs with and without the sample, as well as, the weight of the collection tube before and after sample processing were recorded and used to calculate saliva sample retention and sample recovery rate. The experiment was performed in triplicate.

#### 4.3.4 Preparation of saliva samples for total protein concentration and rheology test

Fifteen milliliter of saliva samples collected from eight healthy individuals were pooled and aliquoted into five aliquots of 3 ml. Each aliquot was treated with different saliva processing technique: (1) Nonprocessed native samples, (2) SHEAR SCD‐processed samples, (3) freeze‐thawing‐processed samples, (4) DTT‐processed sample, and (5) Supernatant (centrifuged samples). For the freeze‐thawing‐processed saliva, the samples were first frozen in −20°C for an hour and subsequently thawed in room temperature for 30 min. DTT (Invitrogen, USA) was reconstituted in ultrapure water to a concentration of 10 mM. The DTT‐processed saliva was obtained by mixing equal amount of 10 mM DTT and saliva. Supernatant samples were extracted after centrifuging saliva in a bench top centrifuge machine (Centrifuge 5810, Eppendorf, Germany) at 3000*g* for 10 min.

#### 4.3.5 Total protein concentration test

BCA protein assay (Thermo Fisher Scientific, IL, USA) was used to estimate the total protein concentration of saliva samples. One milliliter of each type of processed saliva samples (native, SHEAR SCD‐processed, DTT‐processed, freeze‐thawing‐processed, and Supernatant) was extracted and diluted with 2 ml of ultrapure water. The assay was performed in a 96 well plate according to the user manual.^28^ The plate was read with a microplate reader (Spark 10m, Tecan, Switzerland) at 562 nm wavelength. Protein concentration of one freeze‐thawing‐processed saliva sample was above the working range of BCA assay and was removed as an outlier.

#### 4.3.6 Rheology

Viscosity of processed saliva samples (native, SHEAR SCD‐processed, DTT‐processed, freeze‐thawing‐processed and supernatant) and buffer solution were measured with a rheometer (MCR302, Antoon Paar, Austria) over a shear rate range from 50 to 3000 s^−1^ at 25°C using the cone plate measuring system (CP25‐2). The measurement for each sample type was repeated 5–7 times.

### 4.4 Rapid antigen testing procedures

*Panbio™ COVID‐19 Ag Test Kit* (Abbott, USA) was used as the ART kit. Saliva samples of each volunteer were aliquoted into two parts and processed with SHEAR SCD and commercial SCD. Exactly, 4.5 μl of SARS‐CoV‐2 nucleocapsid protein (1 μg/μl)^47^ and 95.5 μl of ART buffer solution were added to 500 μl of each processed saliva sample and a control solution ‐ phosphate buffer saline solution (PBS). The mixture was vortexed for 1 s, and five drops were loaded onto the sample well of the ART cassette. Time‐lapse videos (time‐lapse interval = 10 s) of the development of each cassette was recorded with GoPro Hero 4 (GoPro, USA) mounted within a custom‐made lightbox to provide fixed imaging conditions. The time‐lapse videos were processed with ImageJ to analyze parameters such as line intensity and liquid migration speed (Figures S3,S5 and Video S1).

### 4.5 Pressure measurement

The maximum pressure generated within the funnel of SHEAR SCD during the folding and squeezing process was measured with a pressure transducer (Analog Pressure Sensor, Gravity) inserted at the bottom of the funnel. One milliliter saliva samples from three participants were added in each replicate test.

### 4.6 User study

The user study sought to evaluate the end user's experience of the SHEAR SCD and commercial SCD. Participants were asked to donate 1 ml of saliva to each of the two SCDs. They were subsequently interviewed based on a semi‐structured interview guide (Table 1). The average length of the interview was 21 min (range: 18–25 min). The semi‐structured interviews audio‐recorded, transcribed verbatim, and analyzed using thematic analysis, where the responses were descriptively labeled for primary coding. The labels were then categorized into different groups, which were subsequently used to create broader themes.

**TABLE 1:** List of questions included in the usability study

Focus area/main themes	Examples of questions and probes
SHEAR SCD	What are your thoughts on the devices? Likes and dislikes?
	How do you feel about the process? Ease? Comfortable rolling and squeezing your own saliva? Safe? Intuitive? Did you face any difficulties squeezing the funnel? Was it hard to do so? How confident did you feel using the test device? What do you think of the instructions on how to use the device? What can be improved?
Saliva as a diagnostic material	Would you use this as part of a formal diagnostic test? What might keep you/others from using this device? Have you undergone any COVID‐19 detection test? Which method did you undergo? How does it compare? Have you collected your saliva in a different device before? What device? How does it compare?
User's consideration for the adoption of diagnostic kit with SHEAR SCD	What would stop you from using a device like this? What are the aspects that will affect your decision to purchase a device like this?

### 4.7 Statistical analyses

Statistical analyses were carried out using PRISM 9 (version 9.3.1, GraphPad). Statistical significance was determined using the Kruskal–Wallis test with Dunn's test at *α* = 0.05 for the food particle, rheology and protein test, and Wilcoxon signed‐rank test at *α* = 0.05 for the examination with the ART.

## 5 CONCLUSIONS

We developed an SCD with sample homogenization capabilities that enhances interface for downstream analytical processes. SHEAR SCD‐processed saliva samples exhibited lowered viscosity with enhanced uniformity, increased detectable total protein concentration and an augmented visibility of test line when analyte was spiked at LOD concentration and detected with an ART. Importantly, participants from the user study highlighted the ease of use of SHEAR SCD and preference of saliva collection over collection of some other diagnostic samples. In sum, the strategic implementation of SHEAR SCD may play an important role in enabling rapid and self‐contained saliva processing for diagnostic applications, especially in the context of POC testing during outbreak, epidemic, and pandemic conditions.

## AUTHOR CONTRIBUTIONS

*Conceptualization*: Agata Blasiak, Paul MacAry, Dean Ho. *Methodology*: Agata Blasiak, Shang Wei Song, Paul MacAry, Dean Ho, V Vien Lee, Rashi Gupta, Xinlei Qian, Yue Gu. *Investigation*: Agata Blasiak, Shang Wei Song, V Vien Lee, Yoann Sapanel, Niharika Jothilingam. *Supervision*: Agata Blasiak, Paul MacAry, David Michael Allen, John Eu Li Wong, Dean Ho. *Writing—original draft*: Agata Blasiak, Shang Wei Song, Niharika Jothilingam, Paul MacAry, Dean Ho. *Writing—review and editing*: Agata Blasiak, Shang Wei Song, V Vien Lee, Rashi Gupta, Yue Gu, David Michael Allen, Paul MacAry, Dean Ho.

## FUNDING INFORMATION

Agata Blasiak gratefully acknowledges funding from the RIE2020 Ministry of Education's Decentralised Gap funding under the TAP programme, administered by NUS Enterprise of the National University Singapore (grant number TAP2002020‐05‐21). Dean Ho gratefully acknowledges support from the Office of the President, Office of the Senior Deputy President and Provost, and Office of the Deputy President for Research and Technology at the National University of Singapore. Dean Ho also gratefully acknowledges funding from the Institute for Digital Medicine (WisDM) Translational Research Programme (grant number R‐719‐000‐037‐733) at the Yong Loo Lin School of Medicine, National University of Singapore, Ministry of Education Tier 1 FRC Grant (grant number R‐397‐000‐333‐114), Micron Foundation, and Sun Life Singapore.

## CONFLICT OF INTEREST

Agata Blasiak, Paul MacAry, and Dean Ho are co‐inventors of a pending patent pertaining to the SHEAR SCD (WO2022055417). All other authors declare they have no competing interests.

## Supporting information


**Data S1:** Supporting Information

Video S1:

## Data Availability

All data needed to evaluate the conclusions in the article are present in the article, the Supporting Information and the Supporting Information Video.
