# Supplementary material for: SHEAR saliva collection device augments sample properties for improved analytical performance
Source: Bioeng Transl Med. 2023 Jan 17;8(6):e10490. doi: 10.1002/btm2.10490 (PMC10658560; doi:10.1002/btm2.10490)
Supplement: Supplementary file 1 — Data S1: Supporting Information [file BTM2-8-e10490-s001.docx]

**Supplementary Information**

# Title: SHEAR Saliva Collection Device Augments Sample Properties for Improved Analytical Performance

## Authors

Shang Wei Song^1^, Rashi Gupta^2,3^, Jothilingam Niharika^1^, Xinlei Qian^2,3^,Yue Gu^2^, V Vien Lee^1,4^, Yoann Sapanel^4^, David Michael Allen^5,6^, John Eu Li Wong^5,7^, Paul MacAry^2,3^*, Dean Ho^1,4,8,9^*, Agata Blasiak^1,4,8,9^*

## Affiliations

^1^ The N.1 Institute for Health (N.1), National University of Singapore, Singapore 117456.

^2^ Life Sciences Institute, National University of Singapore, Singapore 117456.

^3^ Department of Microbiology and Immunology, Yong Loo Lin School of Medicine, National University of Singapore, Singapore 117545.

^4^ The Institute for Digital Medicine (WisDM), Yong Loo Lin School of Medicine, National University of Singapore, Singapore 117456.

^5^ Department of Medicine, Yong Loo Lin School of Medicine, National University of Singapore, Singapore 119228.

^6^ Division of Infectious Diseases, National University Hospital, Singapore 119074.

^7^ Department of Haematology-Oncology, National University Cancer Institute, Singapore, National University Hospital 119074.

^8^ Department of Biomedical Engineering, College of Design and Engineering, National University of Singapore, Singapore 117583.

^9^ Department of Pharmacology, Yong Loo Lin School of Medicine, National University of Singapore, Singapore 117600.

*Correspondence: Paul A Macary: micpam@nus.edu.sg, Dean Ho: biedh@nus.edu.sg, Agata Blasiak: agata.blasiak@nus.edu.sg

**This File Includes:**

Supplementary Results

Supplementary Materials and Methods

Figs. S1 to S6

Tables S1 and S2

# Supplementary Results

**SHEAR SCD performance on saliva samples of high viscosity**

The viscosity of human saliva is affected by variables such as the collection method, temperature and interhuman variability. A study revealed that thicker and more viscous saliva was collected from subjects with smoking habits.^1^ To investigate the performance of SHEAR SCD, saliva samples with higher viscosity were measured across a shear range from 50 to 3000 s^-1^ with a rheometer. While no significant differences in saliva viscosity were detected between SHEAR SCD-processed saliva and Native saliva, at the low shear rate (50 s^-1^), we observed a 40% times reduction in median saliva viscosity of SHEAR SCD-processed saliva with improved uniformity (**Figure S6a**). While the median viscosity of SHEAR SCD-processed saliva (2.00 (95% CI, 1.83 - 2.07)) was similar to Native saliva (1.98 cP (95% CI, 1.94 - 4.84)) (**Figure S6b**) at the high shear rate (3000 s^-1^), the viscosity of SHEAR SCD-processed saliva was more uniform with a CV of 0.04 compared CV of 0.41 for native saliva. This suggests the robustness of the mechanical shearing effect for saliva sample homogenization.

### **User’s perspective on saliva as a biological material for diagnostic tests**

All participants reported to have prior experience with diagnostic tests that involve collection of biological samples including urine, blood and nasal swabbing. Four participants had undergone prior saliva-based diagnostic tests which involved either spit collection or/and cheek swabbing. In general, the participants were highly receptive towards saliva as a biological material for diagnostic tests and most had no concerns of processing their own saliva. All participants commented positively on the ease of collection of saliva samples, which was attributed to comfortability (neither painful nor invasive), simplicity of the collection process and high confidence towards self-collection. While half of the participants highlighted their preference for saliva collection over nasal swabbing, some participants had reservations regarding the collection method. Five participants expressed concerns about the safety aspect of saliva collection, citing higher environmental contamination risk due to higher probability of saliva sample spillage during the discarding process when compared to nasal swabbing. In addition, two participants preferred cheek swabbing over expulsion saliva collection. Overall, the comments on saliva collection methods were largely positive suggesting the receptiveness and potential of saliva as a biological material for diagnostic applications (**Table S1**).

### **User’s consideration for the adoption of diagnostic kit with SHEAR SCD**

When participants were asked to list their consideration for the adoption of a diagnostic kit that contains SHEAR SCD, they highlighted the accuracy of the diagnostic kit, cost, hygiene, safety and, ease-of-use as their main considerations. For saliva-related considerations, the recognition status of saliva test by government, the amount of saliva required for accurate testing and the pre-testing requirement of no drinking, eating and brushing of teeth for a time period were some of the participants’ concerns (**Table S1**).

# Supplemental Materials and Methods

## Image processing and analysis for the food particulate test

ImageJ (National Institute of Health, USA) was used to perform image processing and analysis. The images captured by the microscope (ECLIPSE Ti-S, Nikon, Japan) were resized (1280 by 500 pixels) to remove background noise and subsequently converted into 8-bit, greyscale images. Image thresholding of the greyscale images at two ranges (0-125 and 177-255) were conducted to identify both light and dark food particles. The two images were then stitched for the measurement of the size and area of particles were using the analyze particle plugin function (**Figure S4**).

## Image processing and analysis for the ART test

The time-lapse videos were aligned and converted into 8-bit image stacks with ImageJ for signal intensity quantification (**Figure S5b**). The average pixel intensity of the regions of interest at the test line (*I_test line_*) and background (*I_background_*) were measured and test line intensity ratio (*I_test line_* */* *I_background_*) was calculated^2^ at 20 (t_20_) and 30 (t_30_) minutes after the sample loading. Test line formation was analysed through the measurement of the average pixel intensity of the region of interest at the test line and 20 pixels before and after the test line (*I_test line_*, *I_before test line_*, *I_after test line_*, respectively). Test line is determined to be formed if (*I_before test line_* / *I_test line_*) and (*I_after test line_* / *I*_test_ *_line_*) are both more than 1.01 and average pixel intensity of *I_before_* _test line_ and *I_after test line_* is not more than 120 grayscale value (**Figure S5c**). Equations and rules used in the ART analysis are summarized in **Table S2**.

**Supplemental** **Figures**


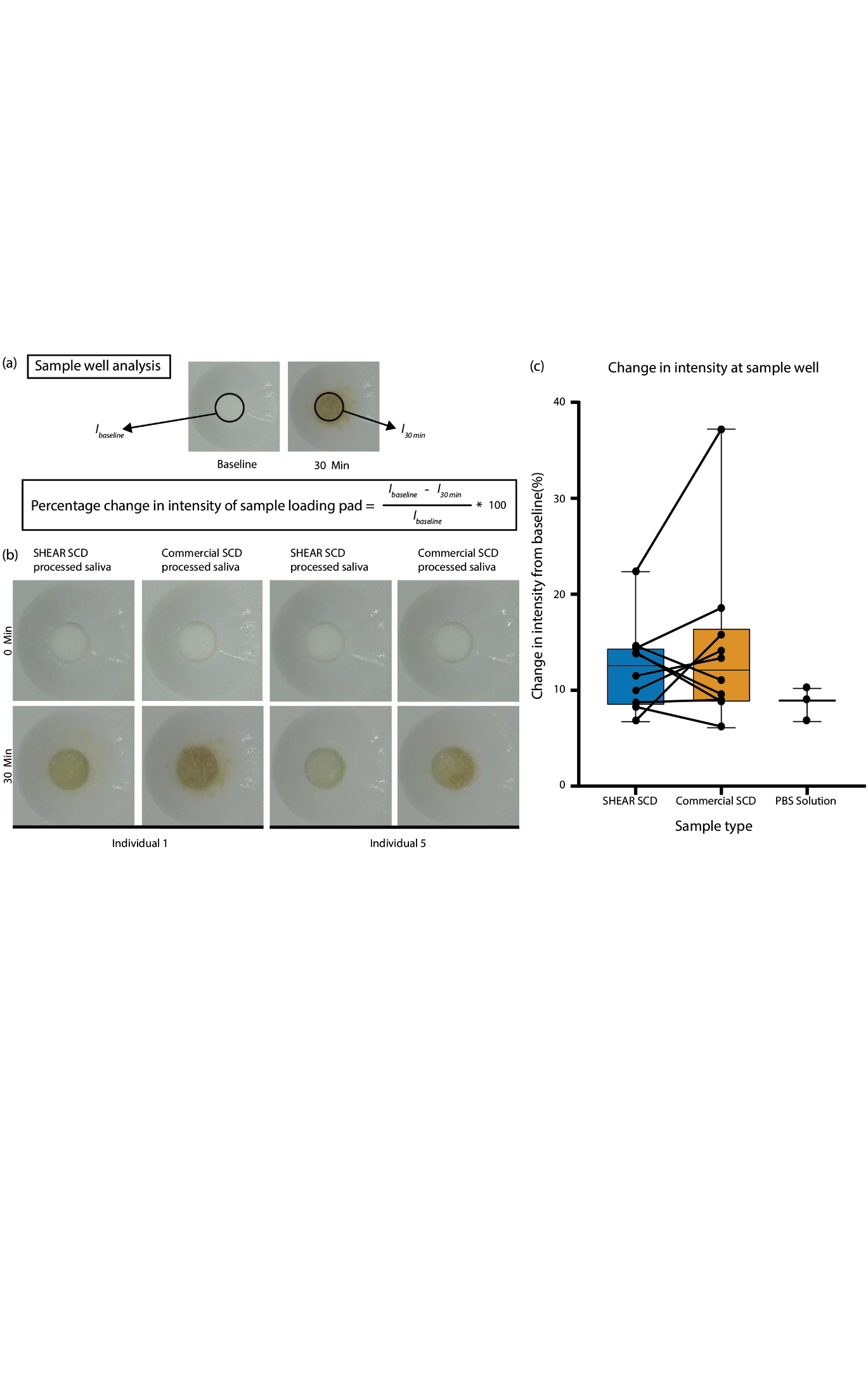


**Fig. S1. Color intensity of sample well.** (**a**). Analysis method of percentage change in color intensity of the sample well. (**b**). Photograph of sample well of participant 1 and 5 at minute 0 and minute 30, before and after loading of the SHEAR SCD-processed and commercial SCD-processed saliva samples respectively. (**c**). Change in color intensity of the sample well of the paired SHEAR SCD-processed saliva (N = 10), commercial SCD-processed saliva (N = 10) samples and, as an independent control, PBS solution (N = 3). N = number of technical replicates. Whiskers represent maximum and minimum values, the box represents the median value, 25^th^ and 75^th^ percentile and the line represent the pairwise comparison between SHEAR SCD-processed saliva and Commercial SCD-processed saliva (c). No statistical difference detected with Wilcoxon signed-rank test at α = 0.05.


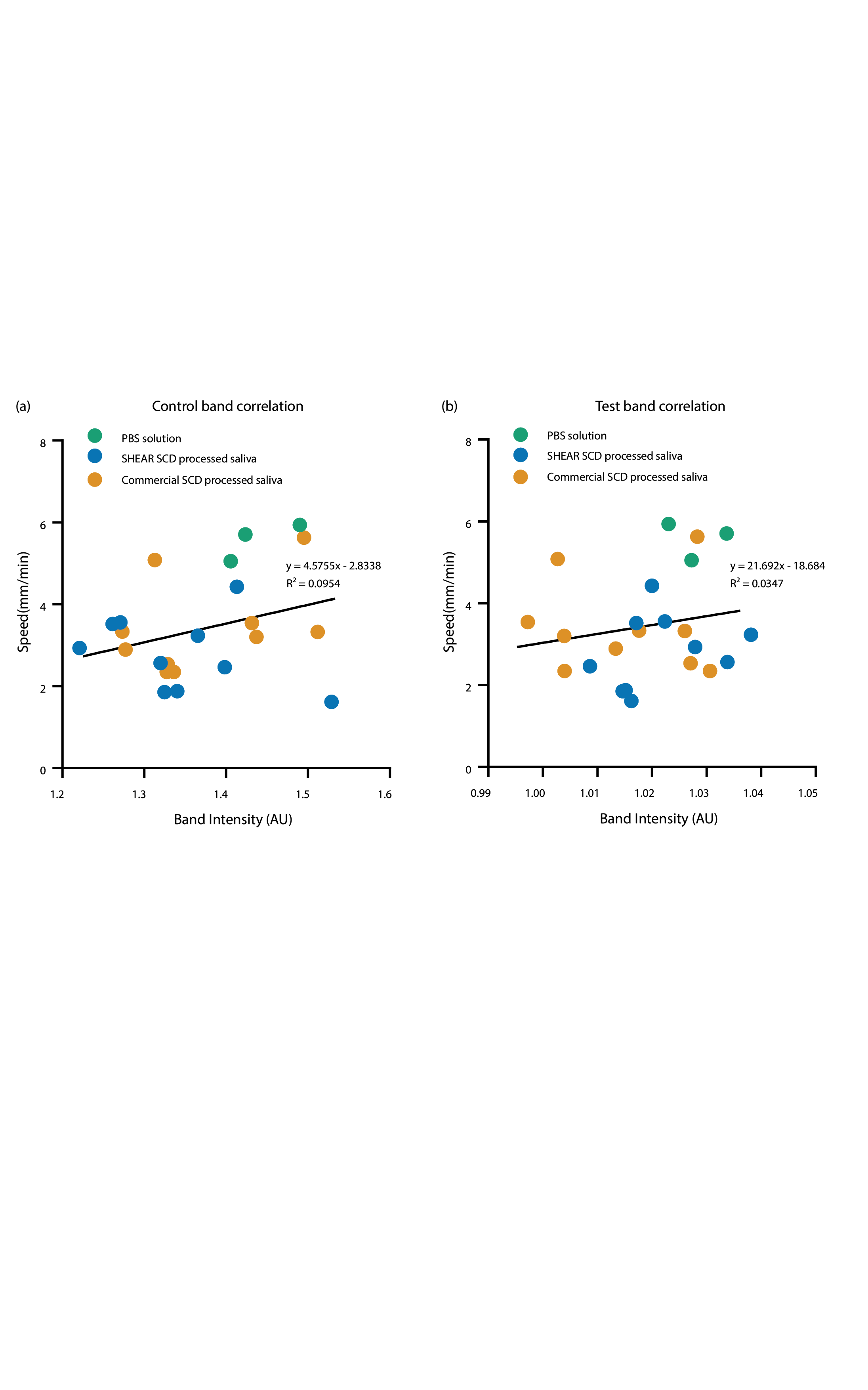


**Fig. S2. Correlation between line intensity and liquid migration speed.** Scatter plot with linear regression of liquid migration speed and intensity of (**a**) test line and (**b**) control line of SHEAR SCD-processed saliva (N = 10), commercial SCD-processed saliva (N = 10) and PBS solution (N = 3). N = number of technical replicates.


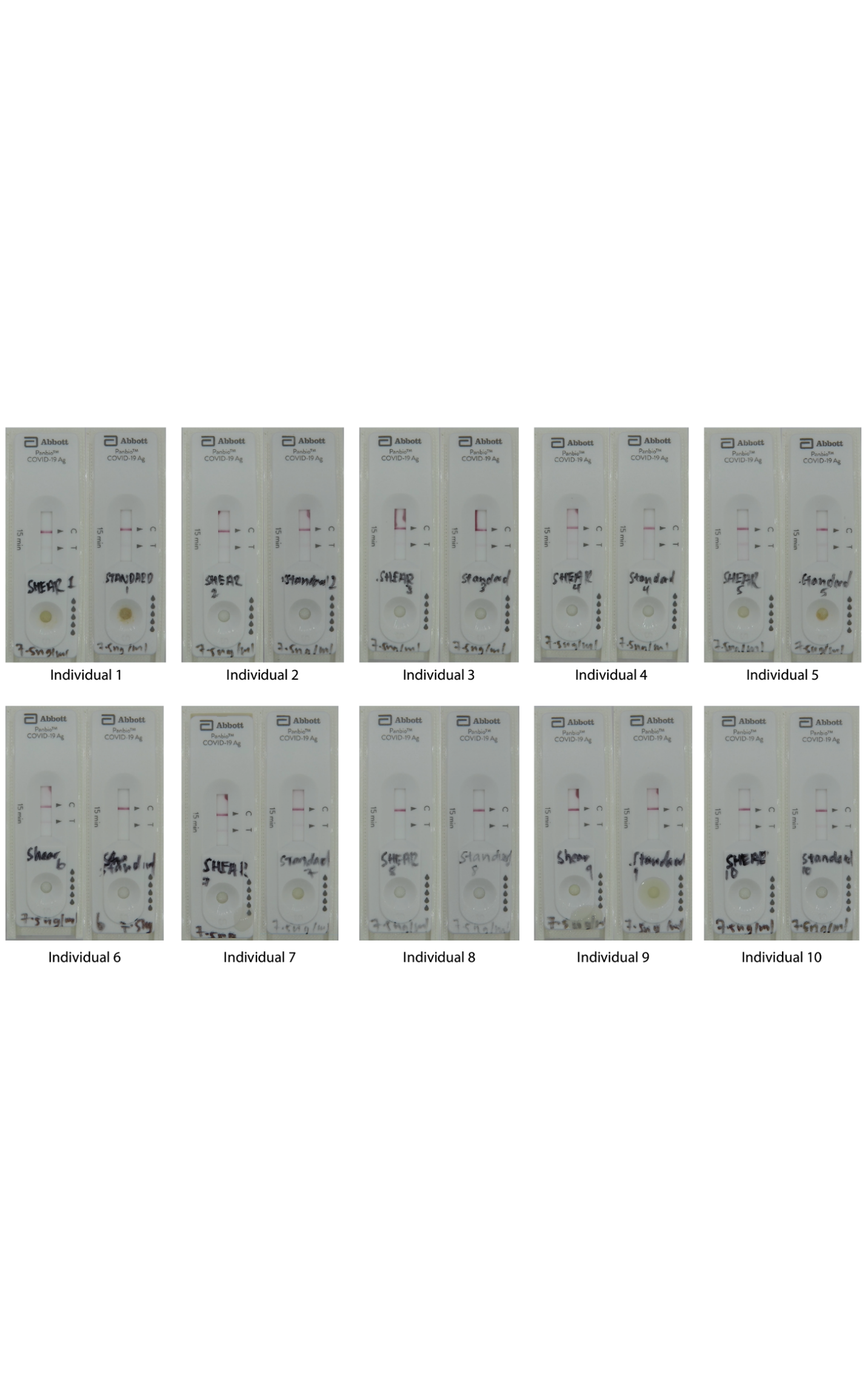


**Fig. S3. Photographs of antigen rapid test cassette used in the ART test.** Photographs of the antigen rapid test cassette captured 30 minutes after the loading of the SHEAR-SCD processed saliva sample and the commercial-SCD processed saliva sample in the sample well from participants (N = 10) of the rapid antigen testing. N = number of technical replicates.

**
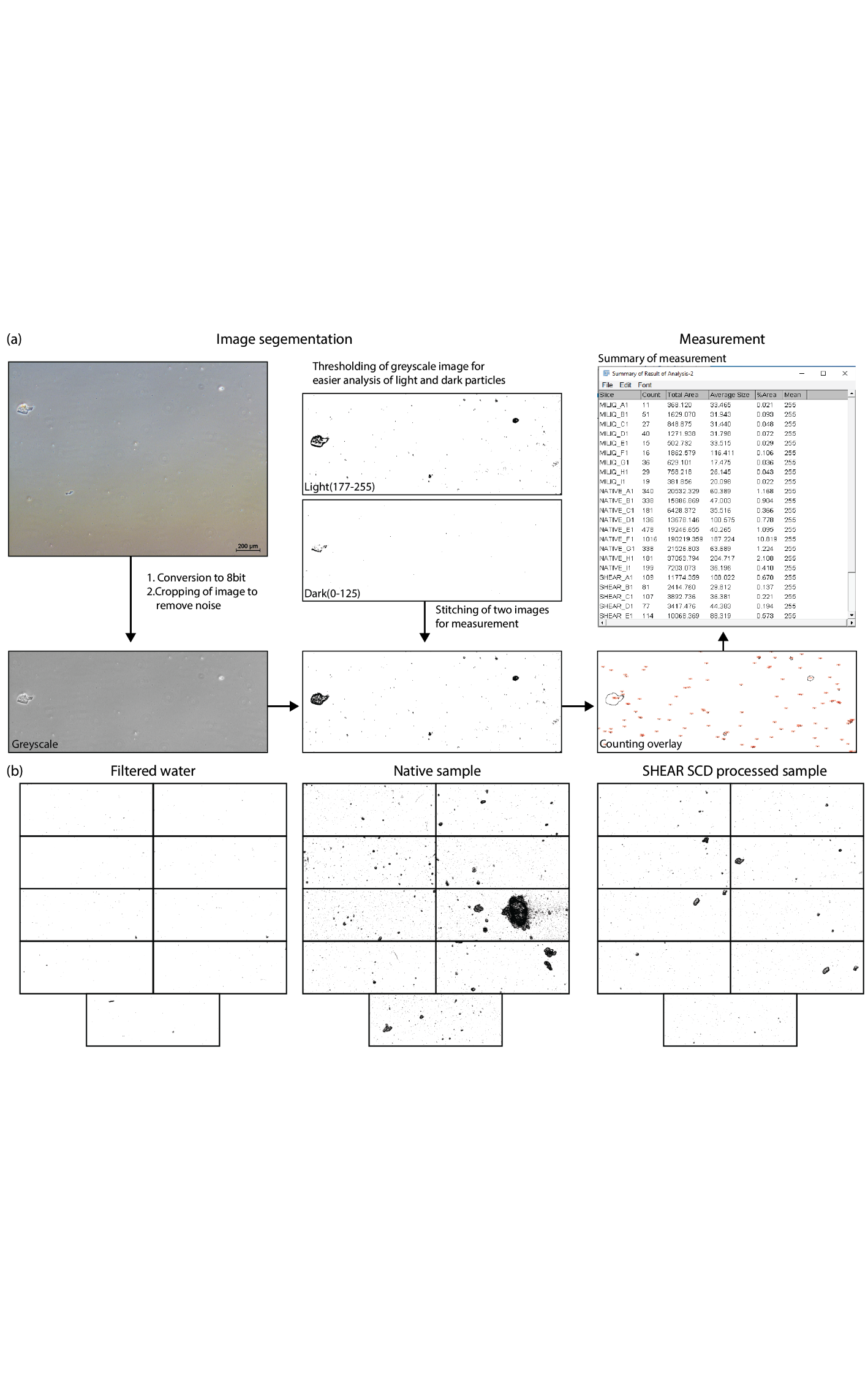
**

**Fig. S4. Image processing food particulate test.** (**a**). Image segmentation method used in ImageJ for the measurement of area and count of food particles present in Filtered water, Native sample and SHEAR SCD processed sample (**b**). Processed images of Filtered water, Native sample and SHEAR SCD processed sample at nine fixed points.

**
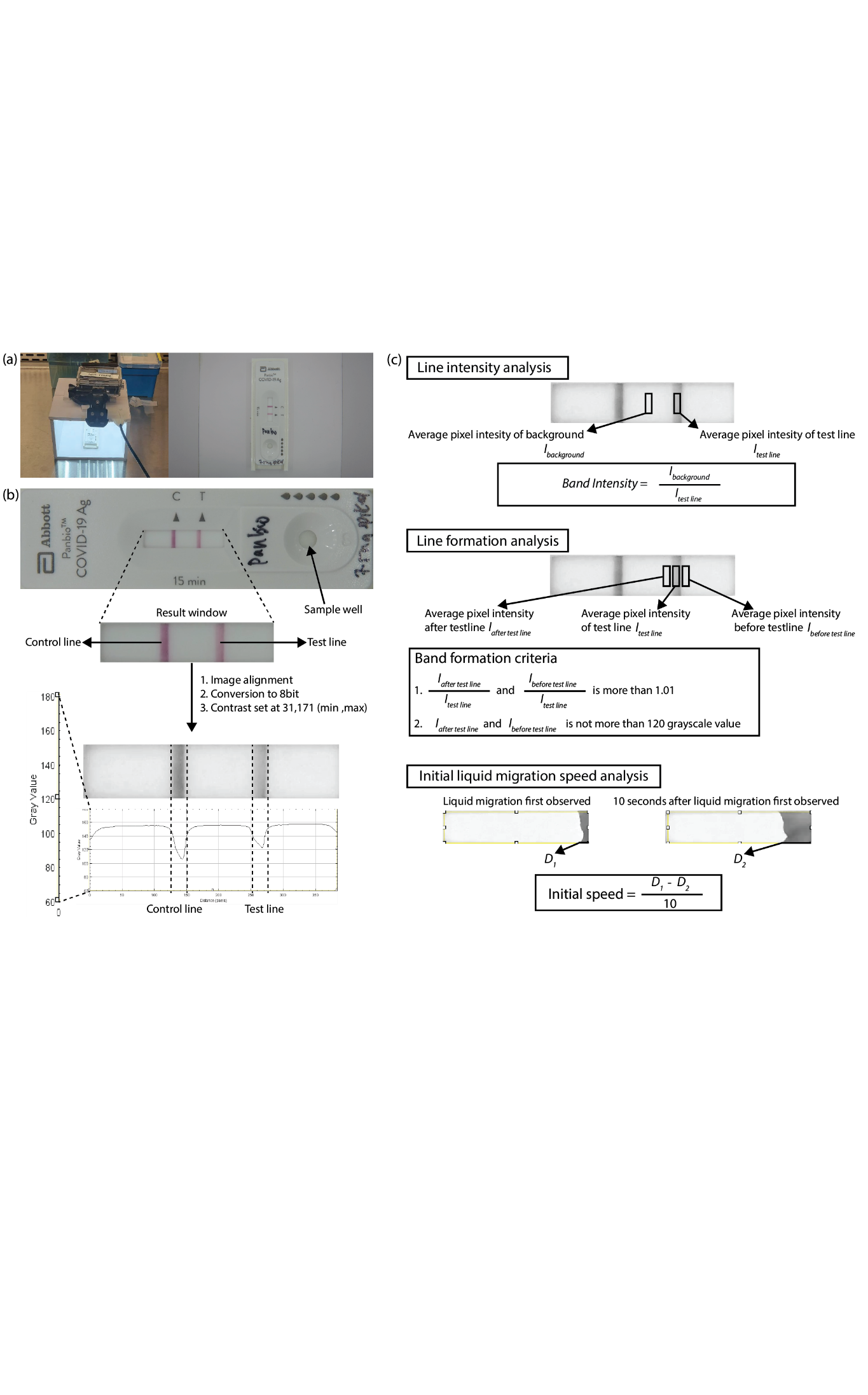
**

**Fig. S5**. **Analysis method for the ART test.** (**a**) Photo of the set-up for the capturing of time-lapse video for the antigen test experiment. (**b**) Antigen test cassette and general image processing step. (**c**) Description of analysis conducted in the antigen test experiment.

**
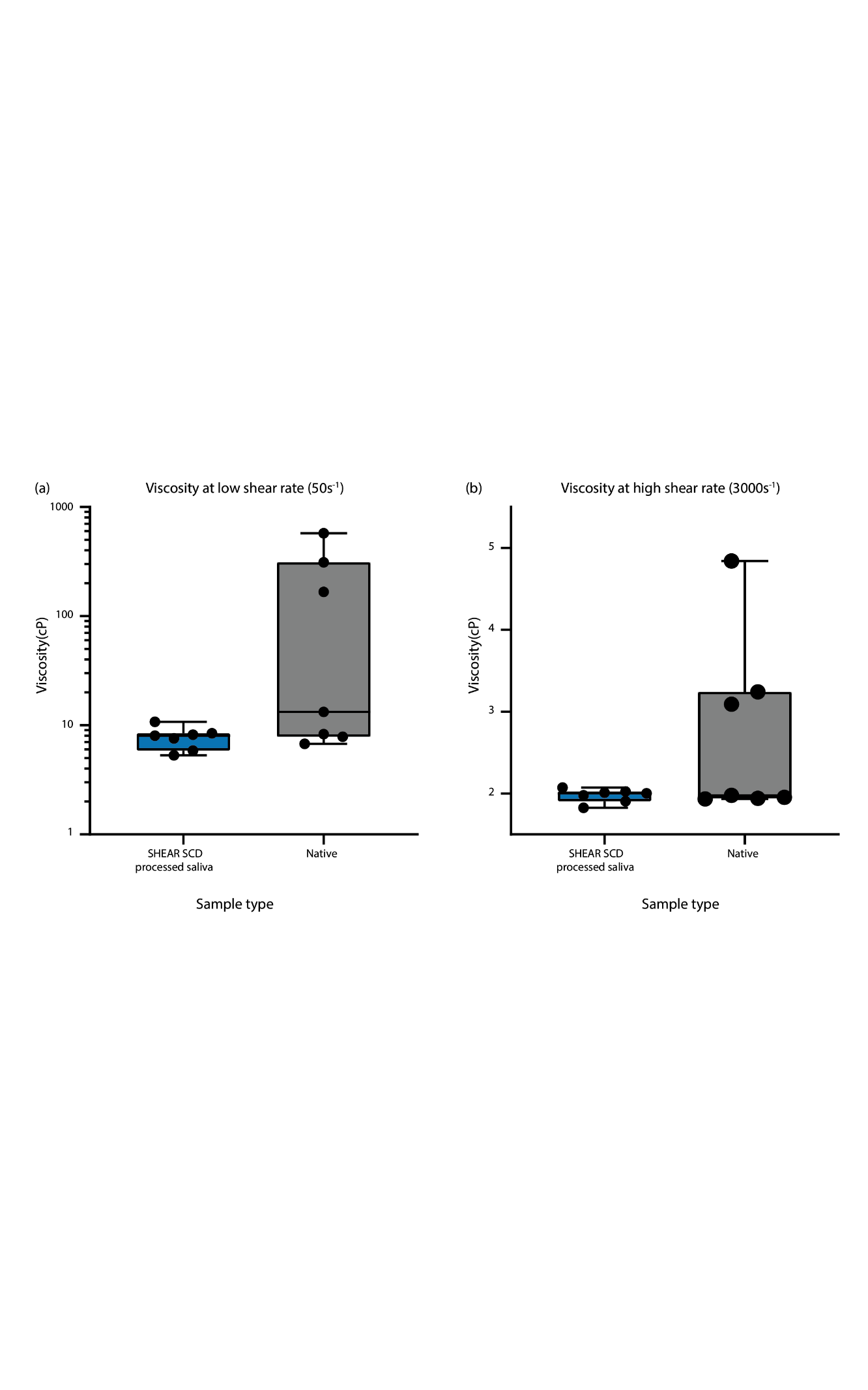
**

**Figure S6** **SHEAR SCD performance on saliva samples of high viscosity.** Viscosity of the SHEAR SCD-processed saliva (N = 7) and Native saliva (Native, N = 7) samples measured with rheometer at the low shear rate: 50 s^-1^ (**a**) and the high shear rate: 3000 s^-1^ (**b**). N = number of technical replicates. Whiskers represent maximum and minimum values and the box represents the median value, 25^th^ and 75^th^ percentile. No statistical difference detected with Mann Whitney U test at α = 0.05.

**Table S1. Example quotes of each focus area from the user study**

| **Focus area 1: Usability & Functionality of the SHEAR SCD** | | |
| --- | --- | --- |
|  | Specifics | Example quote |
| Positive | SHEAR SCD is straightforward and easy to use | “It [SHEAR SCD] was straightforward and it’s easy to use to… in terms of how it functions, it’s not hard to understand.” – Participant 6 |
|  | Wide funnel allows for easy spitting process | “Because there was a much larger surface area, [I] don’t have to worry about trying to fit my mouth into the funnel in order to contribute the saliva.” – Participant 5 |
|  | Wide funnel feels safer due to reduction in saliva spillage | “I think [the SHEAR SCD] is safer because you [spit] closer. You put your mouth closer and you open the flaps. Nothing is missing and going outside.” -Participant 2 |
|  | Process of squeezing and rolling is quick and easy | “It [SHEAR SCD] was really easy, because I thought the saliva wouldn’t go through but I just push it and it all went in 1 move. So, it was quite fast and efficient.” – Participant 7 |
|  | Process of squeezing and rolling allows for clearer indication of saliva level | “I thought that the rolling of the bag down to secrete the saliva down into the tube was quite interesting. I could see that after folding the bag and as I watch the saliva flow down, [I] could see the clear distinction between before being push and what’s after; there are less bubbles.” Participant 9 |
|  | SHEAR SCD easier to use for elderly | “I find device 1 is better for those senior citizens because err, it can avoid spillage of the saliva” – Participant 12 |
|  | Clear Instructions | “I think it is quite clear, once you go through the video, you are able to I mean know what to do with the device” Participant 14 |
|  | Processing saliva with SHEAR SCD reduces air bubbles | “I could see that after folding the bag [funnel], and as I watch the saliva flow down, could see the clear distinction between before being push and what’s after; there are less bubbles.” Participant 9 |
| Negative | Commercial SCD more efficient and easier to use | “The [commercial] device is more straightforward because it is just like an opening and closing. You don’t really need to squeeze it out but I was thinking if it is because the [SHEAR] device [has] a filter or something.” – Participant 1 |
|  | Difficulty with flap during the rolling process | “When I was doing the folding down part [the process] was a bit hard, so I just force it down. It didn’t feel like there was a per… not perforation.” – Participant 7 |
|  | Difficulty fully opening funnel | “When you pull [the funnel] open, the top will open but the bottom doesn’t open fully. So, the saliva gets stuck at somewhere in the middle where it open.” – Participant 4 |
|  | Uncertain about amount of saliva needed | “It wasn’t very clear for me the extent to which [the saliva] was [dripping] down into the tube, so I didn’t know if I needed to add more saliva into the upper portion the funnel.” – Participant 6 |
|  | Unsealed funnel before rolling process | “I had trouble sealing the bag. I don’t know if it’s the… it just didn’t seal, so I just folded it.” – Participant 7 |
|  | Backflow of saliva during the squeezing and rolling process | “The [SHEAR] device, there is a degree of backflow if you press too hard. I think there is too much air pressure inside the tube.” Participant 10 |
|  | People with disabilities might find SHEAR SCD hard to use | “Some people particularly those who maybe are have disabilities, it might be quite hard to use” Participant 11 |
| **Focus area 2: Saliva as a biological material for diagnostic tests** | | |
| Positive | Increased comfort level | “It’s [saliva collection] more comfortable than the nasal swab because that hurts” Participant 4 |
|  | Saliva collection is simple to use | “Saliva [collection] will be the easiest and the most convenient method “Participant 10 |
|  | Bleeding after swabbing | “But then the PCR swab was just uncomfortable and then I bleed afterwards, after the nurse poke my nose. It was not a good experience” Participant 10 |
|  | Sneezing after nasal swab | “I would say that it is very uncomfortable to do the nasal swab because I will just keep sneezing after that” Participant 7 |
|  | Prefers saliva collection over current collection methods | “I would definitely choose the saliva collection for sure, for the convenience, not too sure about the cost, but definitely how less invasive it is” Participant 3 |
| Negative | Safety concerns for saliva collection method | “But then the saliva is like you are putting… there is a risk of spillage, that’s probably where my concern is.” Participant 4 |
|  | Prefer Cheek swab over saliva collection | “If I have a choice, I will rather do the cheek swab than do the saliva collection” Participant 7 |
|  | Amount of saliva required for test | “I mean the only thing that I don’t like about these procedures is you have to get a lot of saliva out of your throat” Participant 2 |
| **Focus area 3: User’s consideration for the adoption of diagnostic kit with SHEAR SCD** | | |
| SCD Consideration | Accuracy | “If it is not more accurate, it wouldn’t motivate me to use it. I would be like just give me anyone and I just use any one” Participant 7 |
|  | Cost | “Price point relative to the ART test kits, as long as it doesn’t stray too far, slightly expensive, I think it is worth the trade-off for being uncomfortable.” Participant 9 |
|  | Safety | “So, I guess mainly safety and … so that they are confident that it will work and will be safe and will give a real result.” Participant 2 |
|  | Ease of use | “If I really have to choose between device, I care more about how easy it is to use, like how many steps” Participant 6 |
|  | Hygiene | “I think the factors that would influence my… influence my preference to use this would include things like hygiene” Participant 15 |
| Saliva test requirements | Amount of saliva required | “Depends on the amount of saliva required. Saliva is preferred over swab test if no more than 1ml is required” Participant 11 |
|  | Pre-testing requirements | “Then, I will probably have to figure out, like actually plan in advance when I want to take the test because it also includes even if I take a small snack, (inaudible 20:37) I can’t eat a small snack or brush my teeth, so when can I do it” Participant 5 |

**Table S2**. Equations and terminology in the analysis of Rapid antigen test experiment.

| Test line intensity | *I_background_ / I_test line_* |
| --- | --- |
| Control line intensity | *I_background_ / I_control line_* |
| Line formation | Line formation if   1. *I_before test line_* / *I_test line_* is more than 1.01 2. *I_after test line_* / *I_test line_* is more than 1.01 3. *I_before test line_* and *I_after test line_* $($is not more than 120 arbitrary units) |
| Migration timepoint | Liquid migration first observed |
| D_1_ | Distance travelled at migration timepoint |
| D_2_ | Distance travelled 10 seconds after migration timepoint |
| Initial liquid migration speed | D_1_ - D_2_/10 |
| Loading time | Migration Timepoint – (Initial liquid migration speed / $D_{1}$) |

# References

1. Petrušić N, Posavac M, Sabol I, Mravak-Stipetić M. The Effect of Tobacco Smoking on Salivation. *Acta Stomatol Croat*. 2015;49(4):309-315. doi:10.15644/asc49/4/6

2. Park J. An Optimized Colorimetric Readout Method for Lateral Flow Immunoassays. *Sensors (Basel)*. Nov 22 2018;18(12)doi:10.3390/s18124084
